# The role of SIRT3 in regulating cancer cell metabolism

**DOI:** 10.1186/1753-6561-6-S3-P18

**Published:** 2012-06-01

**Authors:** Karina N Gonzalez Herrera, Lydia W Finley, Marcia C Haigis

**Affiliations:** 1 Department of Cell Biology, Harvard Medical School, Boston, MA 02115, USA; 2 The Paul F. Glenn Labs for the Biological Mechanisms of Aging, Harvard Medical School, Boston, MA 02115, USA; 3 Cancer Biology and Genetics Program, Memorial Sloan-Kettering Cancer Center, New York, NY 10065, USA

## Background

Sirtuins are a family of NAD^+^-dependent deacetylase, deacylase, and/or mono-ADP ribosyltransferase enzymes involved in regulation of many biological processes. Mammals contain seven sirtuins, three of which are localized to the mitochondria (SIRT3-5). SIRT3 has been shown to be the major mitochondrial deacetylase that regulates metabolic enzymes and promotes oxidative metabolism and energy production. Loss of one copy of the SIRT3 gene is observed in various human cancers. Thus, we examined the role of SIRT3 in regulating metabolism in cancer cells.

## Materials and methods

We have examined glucose uptake and lactate production in a variety of human cancer cell lines, as well as wild-type and SIRT3 null mouse embryonic fibroblasts (MEFs). In addition, we have utilized steady-state metabolomics to determine the metabolic profile of wild-type and SIRT3 null MEFs. Lastly, we utilized a combination of techniques, including quantitative RT-PCR and Western blotting, to examine the mechanism by which SIRT3 regulates cancer cell metabolism.

## Results

Our data show that loss of SIRT3 increases glucose uptake and lactate production. Based on these and previous results from our laboratory, SIRT3 functions as a tumor suppressor to repress the Warburg effect by decreasing reactive oxygen species and destabilizing HIF1α [1]. We are currently examining other metabolic pathways important to cancer that may be regulated by SIRT3.

## Conclusions

In conclusion, we show that loss of SIRT3 results in increased glycolysis, which is the metabolic reprogramming observed in some cancer cells and is also known as the Warburg effect.
